# A 10-Year Retrospective Study of Bacterial Infections in a Japanese Regional Hospital: The Impact of COVID-19 and an Earthquake

**DOI:** 10.7759/cureus.82737

**Published:** 2025-04-21

**Authors:** Takuya Sakamoto, Tomoyuki Ushimoto, Junji Moriya, Haruki Takikawa, Shinji Akitomi, Tsugiyasu Kanda, Yuji Kasamaki

**Affiliations:** 1 Department of Pharmacology, Kanazawa Medical University, Uchinada, JPN; 2 Department of Emergency Medicine, Kanazawa Medical University, Uchinada, JPN; 3 Department of General Medicine, Kanazawa Medical University, Uchinada, JPN; 4 Department of General Medicine, Kanazawa Medical University Himi Municipal Hospital, Himi, JPN; 5 Department of Community Medicine, Kanazawa Medical University, Uchinada, JPN

**Keywords:** antimicrobial bacteria, covid-19, earthquake, inpatients, legionella, outpatients

## Abstract

Bacterial infections are a leading cause of patient mortality, and antimicrobial resistance continues to pose a growing global health and economic threat. The combined effects of the COVID-19 pandemic and earthquakes may have influenced patterns of bacterial infections. This study analyzed data on antimicrobial-resistant and susceptible bacteria over a 10-year period (2015-2024), encompassing these natural disasters. During the COVID-19 pandemic, the number of positive bacterial cultures declined and continued to decrease following the earthquake. This trend included antimicrobial-resistant bacteria, such as methicillin-resistant *Staphylococcus aureus* and methicillin-resistant *Staphylococcus epidermidis* (MRSE). Notably, MRSE cases significantly declined during the pandemic period. In addition, infections caused by drug-sensitive *Streptococcus pneumoniae* among inpatients and *Pseudomonas aeruginosa* among outpatients showed a significant reduction, with both continuing to decline in the post-pandemic period. In contrast, *Legionella* infections significantly increased in both the number and frequency of positive cases, a trend that persisted after the 2024 earthquake. These findings differ from previously reported global data. In Japan, widespread mask usage continued even after the pandemic subsided. The results underscore the importance of improving infection control equipment and promoting individual awareness of infection prevention to reduce the risk of future bacterial infections.

## Introduction

Bacterial infections remain one of the leading causes of morbidity and mortality worldwide, significantly contributing to the global healthcare burden. According to WHO, more than 13 million people die each year from infectious diseases, with bacterial infections accounting for a substantial proportion of these deaths [1]. The growing prevalence of antimicrobial resistance has further intensified this burden, complicating the treatment of common infections and resulting in longer hospital stays, increased healthcare costs, and higher mortality rates [2,3].

The COVID-19 pandemic, which began in 2020, has further exacerbated these challenges by disrupting healthcare infrastructure, delaying treatment for non-COVID-related conditions, and altering bacterial transmission patterns [4]. At the same time, natural disasters such as earthquakes can worsen the situation by damaging public health infrastructure, particularly water systems and sanitation facilities, thereby creating conditions that facilitate the spread of bacterial infections [5]. For instance, the 2024 Noto Peninsula earthquake in Japan significantly disrupted local healthcare services, presenting substantial challenges to infection control measures [6].

Despite these overlapping threats, the combined impact of pandemics and natural disasters on bacterial infection patterns remains underexplored. Long-term surveillance studies that examine the effects of both types of public health crises, such as the COVID-19 pandemic and natural disasters, on bacterial infection trends are notably lacking. Existing literature has primarily focused on either pandemic-related or disaster-related infections, with few studies offering a comprehensive, long-term analysis encompassing both.

The objective of this study was to analyze 10 years of bacterial culture data (2015-2024) from a regional hospital in Japan to evaluate trends in both antimicrobial-resistant and susceptible bacterial infections. The targeted bacteria included methicillin-resistant *Staphylococcus aureus *(MRSA), methicillin-resistant *Staphylococcus epidermidis *(MRSE), extended-spectrum β-lactamase-producing *Escherichia coli *(ESBL-producing *E. coli*), *Streptococcus pneumoniae*, *Pseudomonas aeruginosa*, *Legionella*, and *Clostridioides difficile*. Our aim was to assess how infection patterns shifted before, during, and after the COVID-19 pandemic and the 2024 Noto Peninsula earthquake, offering valuable insights into the combined effects of these two major public health crises.

## Materials and methods

Bacterial isolates, including both infections and colonizations, were collected at Kanazawa Medical University Himi Municipal Hospital from 2015 to 2024. Data included the number of patients and the isolation rate for each bacterial species, including specific antimicrobial-resistant strains. Samples were obtained from various clinical sources -blood, sputum, urine, and wound sites - and processed in the hospital’s clinical laboratory. Each bacterial species isolated from a single site was counted individually, even if multiple bacteria were present at the same location.

Inclusion criteria encompassed all clinical specimens collected from both inpatients and outpatients during the study period, as long as the samples produced clearly identifiable bacterial isolates. Specimens that were heavily contaminated or yielded inconclusive bacterial identification results were excluded.

The bacterial species analyzed in this study were selected to comprehensively and objectively assess trends in both antimicrobial-resistant and drug-susceptible bacteria under the exceptional circumstances created by the COVID-19 pandemic and the 2024 earthquake. The selection was based on their clinical relevance and inclusion in standard hospital surveillance protocols, allowing for a representative overview of bacterial infection dynamics during this unprecedented period.

Each year, the number of positive bacterial cases and their proportions relative to total bacterial infections were recorded. Antimicrobial-resistant bacteria - MRSA, MRSE, and ESBL-producing *E. coli *- were analyzed. We compared the number and frequency of positive results among total bacterial isolates across three time frames: before the COVID-19 pandemic (2015-2019), during the pandemic (2020-2023), and in 2024, the year of the Noto Peninsula earthquake.

Similarly, we assessed trends in drug-susceptible bacteria, including *S. pneumoniae *and *P. aeruginosa*, across the same time periods. The analysis also included a breakdown by inpatient and outpatient status, with particular attention to whether individuals had COVID-19. Data from 2024 were included in this evaluation.

*Legionella *infections and *C. difficile *were identified using commercially available diagnostic kits.

## Results

**Figure 1:**
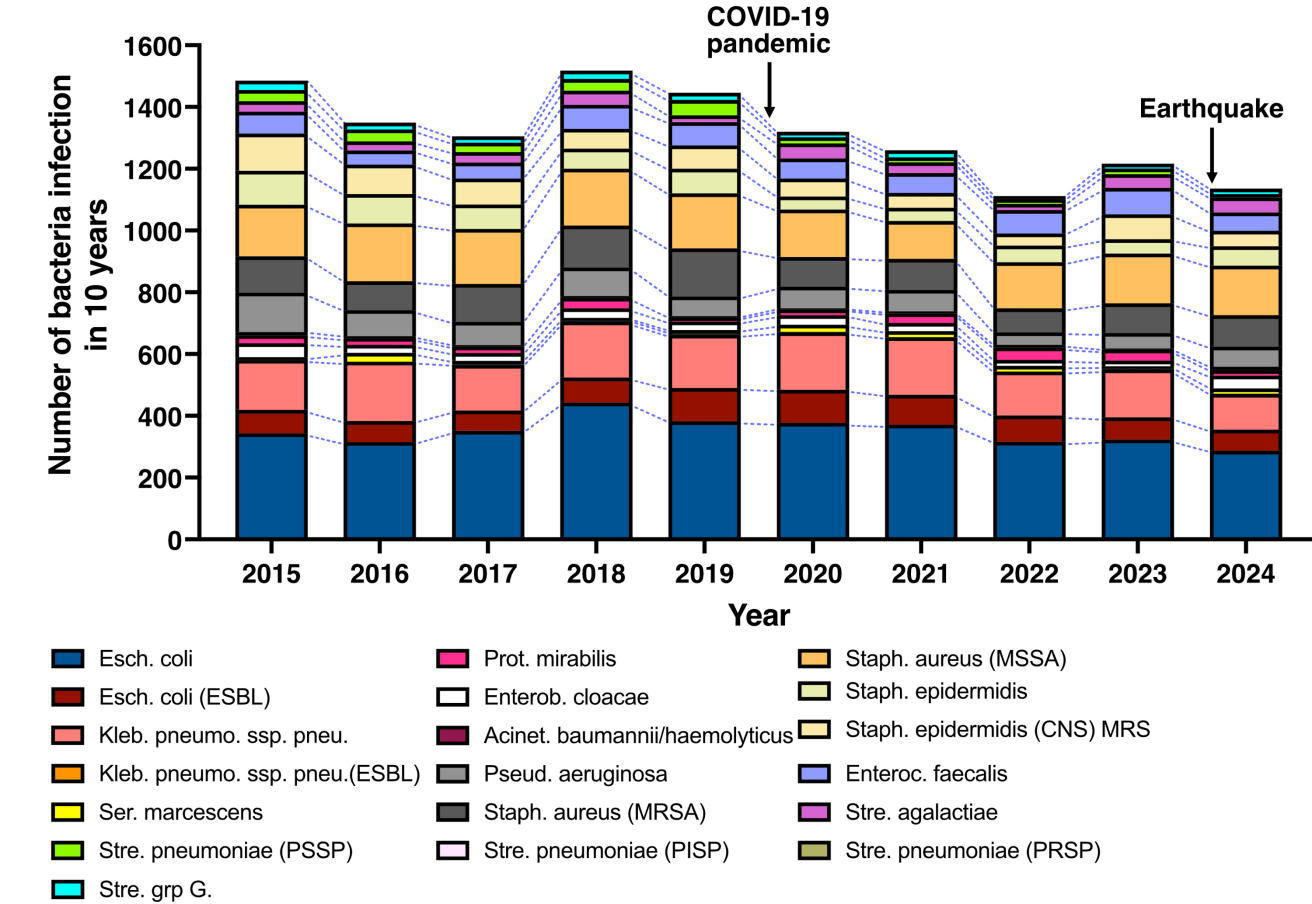
Overall trend in the number of bacterial isolations from 2015 to 2024

The total number of bacteria identified through culture from 2015 to 2024 is shown in Figure 1.

**Figure 2:**
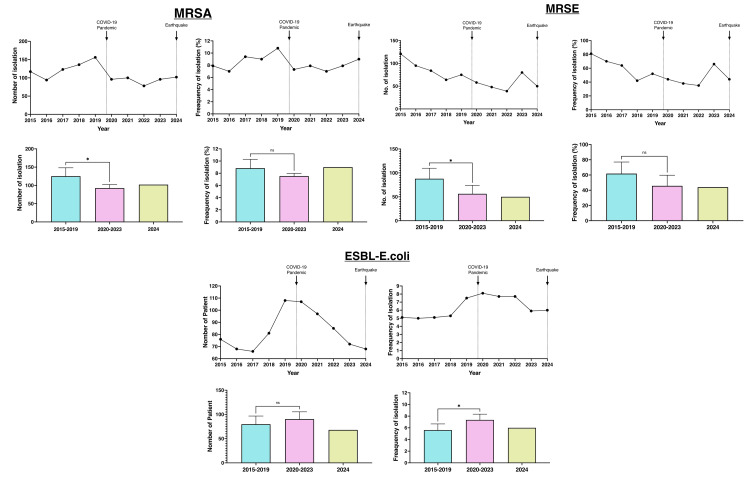
Annual numbers and frequencies of antimicrobial-resistant bacteria The solid arrow marks the onset of the COVID-19 pandemic, while the dotted arrow indicates the 2024 earthquake. “No.” refers to the number of bacterial isolates per year. Frequency indicates the proportion of each bacterial species relative to the total number of isolates. Asterisks (*) denote a statistically significant p-value of <0.05. N-values were not statistically significant.

Compared to the pre-COVID-19 period, the total number of bacterial infections declined following the onset of the pandemic, and this downward trend continued after the 2024 earthquake. Annual trends in antimicrobial-resistant bacteria, including MRSA, ESBL-producing *E. coli*, and MRSE, were analyzed (Figure 2).

MRSA infections significantly declined in number during the pandemic; however, the change in frequency was not statistically significant. For ESBL-producing *E. coli*, although the absolute number of isolates did not significantly change, their frequency increased significantly. In the case of MRSE, the number of isolates decreased post-pandemic, though no significant difference was observed in frequency.

**Figure 3:**
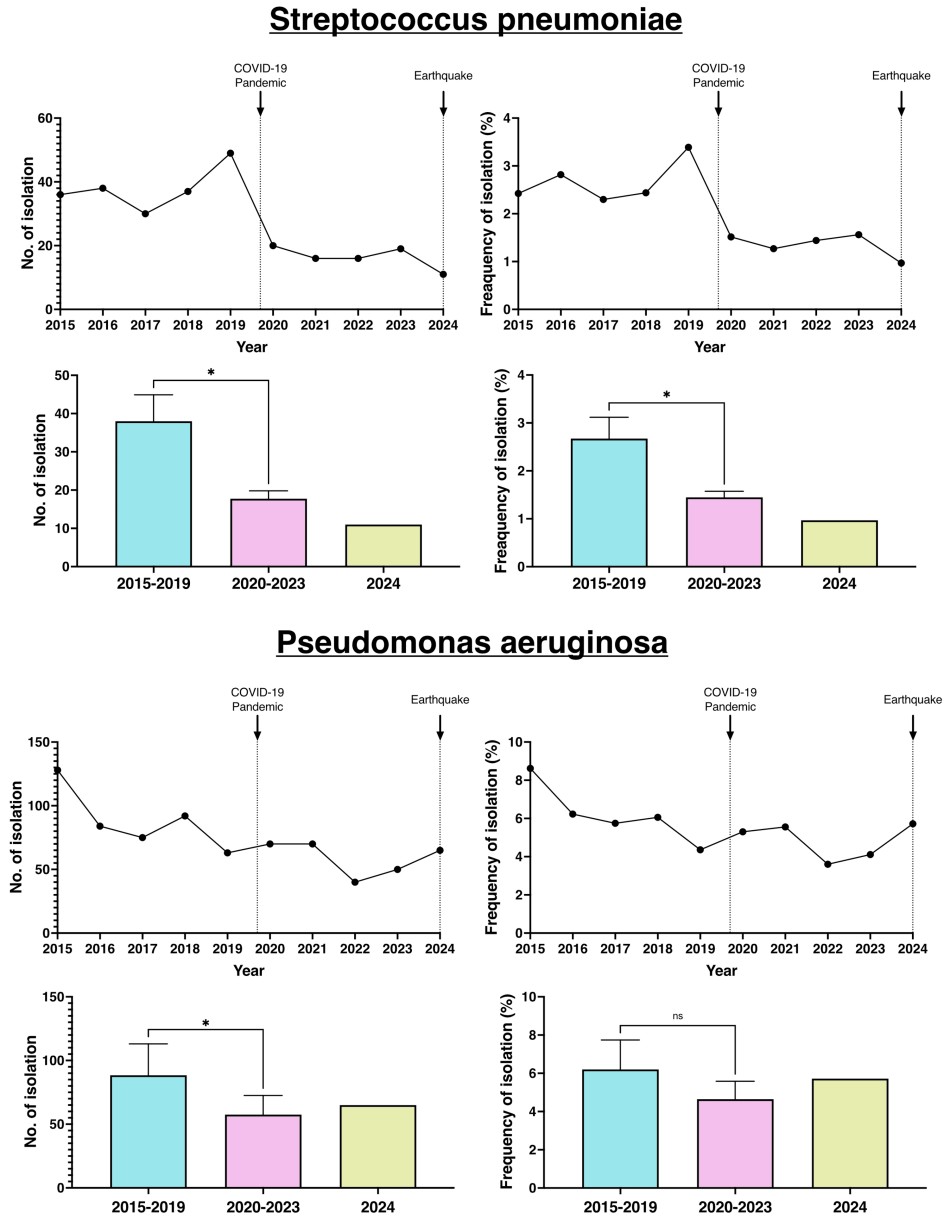
Annual numbers and frequencies of antimicrobial-susceptible bacteria

Antimicrobial-susceptible bacteria - specifically *S. pneumoniae *and *P. aeruginosa *- were also compared between the pre-COVID-19 period and the pandemic years (Figure 3).

*S. pneumoniae *infections showed a significant reduction in both the number of positive cases and their frequency during the COVID-19 pandemic, with this trend continuing even after the earthquake. *P. aeruginosa *showed a significant reduction in the number of positive cases, although the change in frequency was not significant.

**Figure 4:**
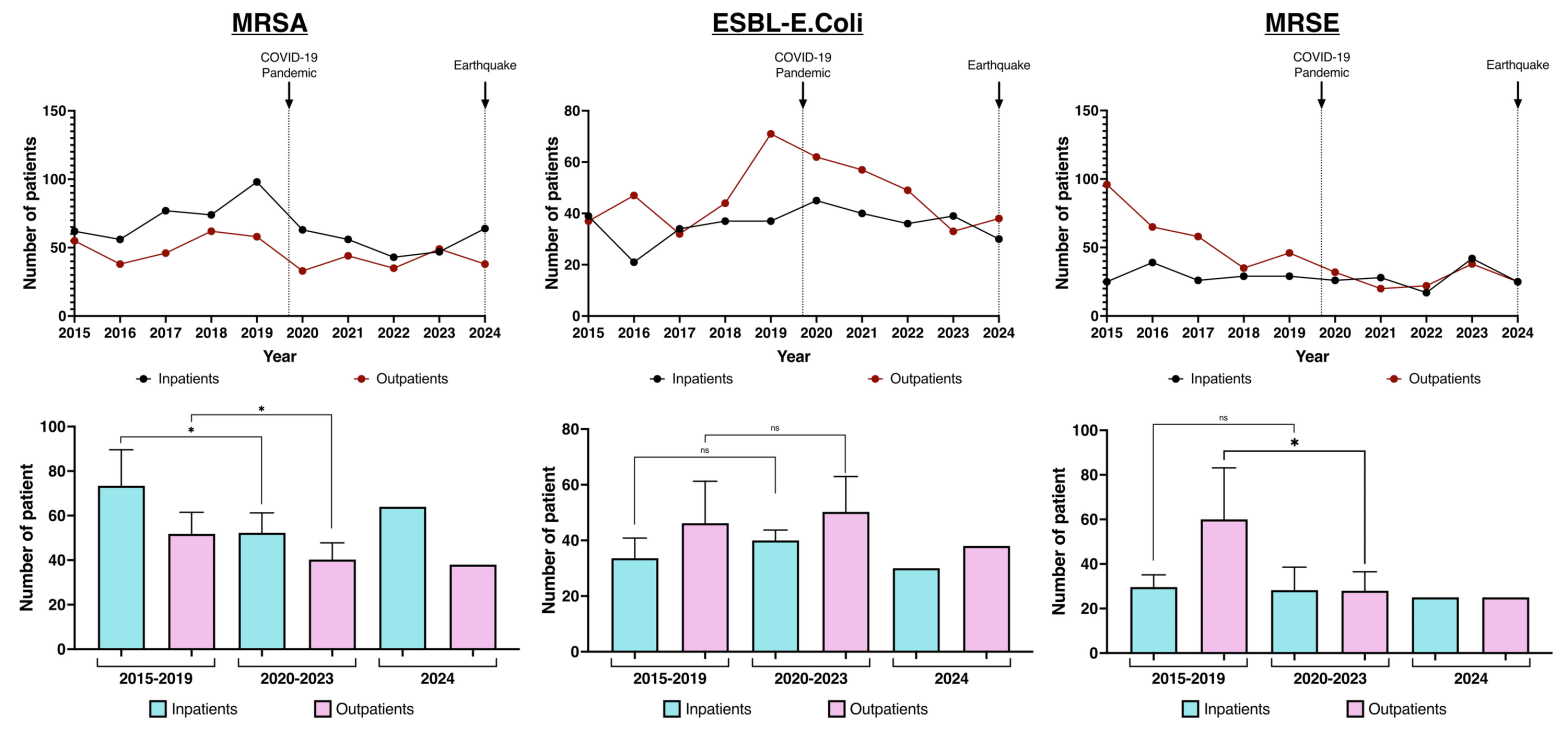
Numbers of inpatients and outpatients with antimicrobial-resistant bacteria

When patients were analyzed by hospitalization status, MRSA infections significantly decreased in both inpatients and outpatients during the pandemic (Figure 4).

**Figure 5:**
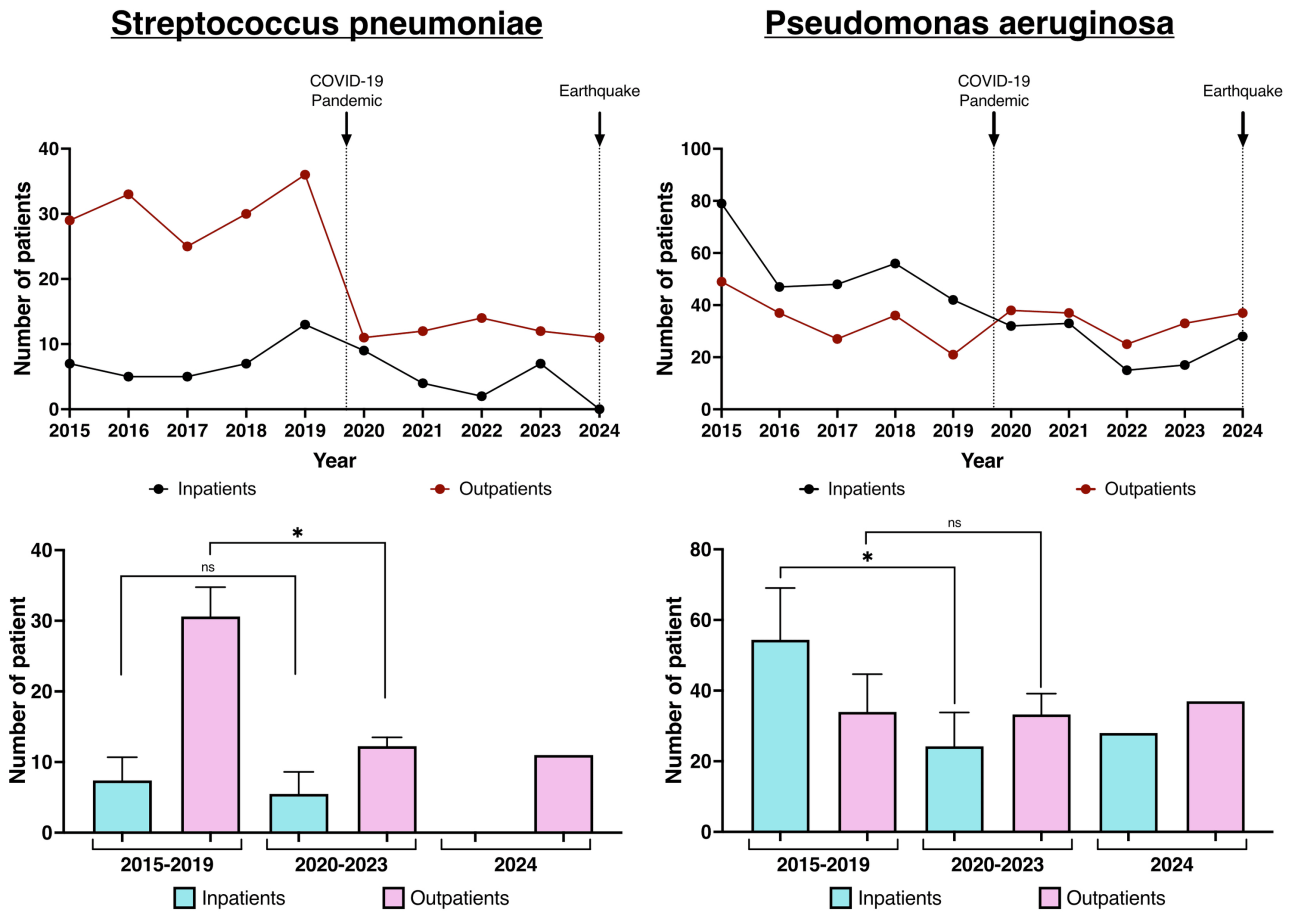
Numbers of inpatients and outpatients with antimicrobial-susceptible bacteria

There were no significant changes in either the number or frequency of ESBL-producing *E. coli *infections. MRSE cases significantly declined among outpatients but remained stable among inpatients. For antimicrobial-susceptible bacteria, *S. pneumoniae *infections significantly decreased in inpatients, while *P. aeruginosa *infections showed a significant reduction among outpatients (Figure 5).

**Figure 6:**
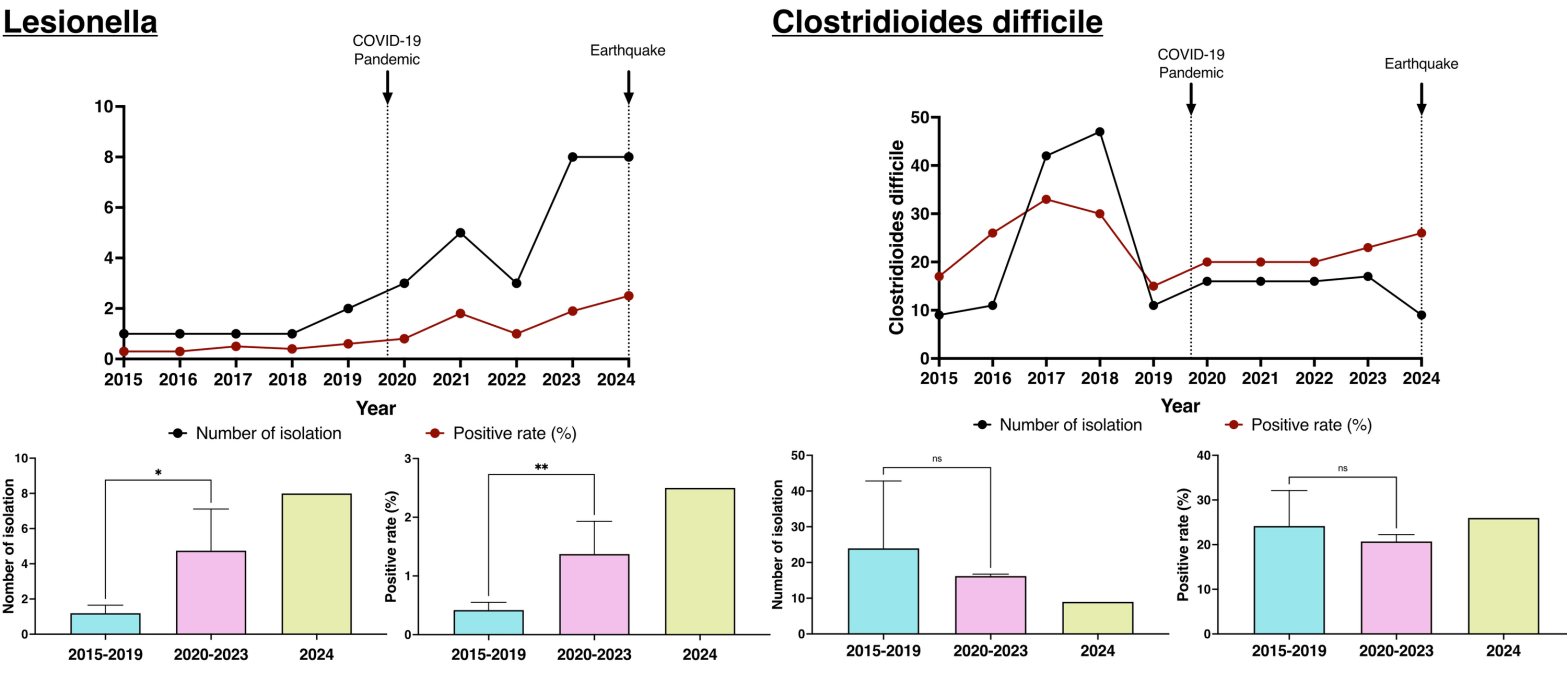
Annual numbers and frequencies of Legionella and Clostridioides difficile

Analysis of *Legionella *infection and *C. difficile *using the Quick Kit revealed a significant increase in both the number and frequency of *Legionella*-positive cases (Figure 6), with this trend continuing after the 2024 earthquake. In contrast, *C. difficile *showed no significant changes before or during the COVID-19 pandemic.

## Discussion

We analyzed changes in bacterial identification over a decade, comparing the period before and after the COVID-19 pandemic, including the effects of the 2024 earthquake. The number of positive bacterial cultures declined due to the COVID-19 pandemic and continued to decrease after the earthquake, including cases of antimicrobial-resistant bacteria such as MRSA and MRSE. Notably, the number of positive MRSE occurrences significantly reduced during the pandemic. While ESBL-producing *E. coli *did not decrease in number, its frequency within the overall bacterial count increased. MRSA infections decreased in both inpatients and outpatients, while MRSE infections saw a significant decline among outpatients. The lack of a significant reduction in ESBL-producing *E. coli *may be attributed to its primary transmission route being contact-based [7], whereas MRSA and MRSE spread through both contact and droplet transmission [8,9]. The reduction in droplet-transmitted infections can likely be attributed to widespread mask use during the pandemic. Additionally, drug-sensitive *S. pneumoniae *and *P. aeruginosa *were significantly reduced in inpatients and outpatients, respectively. The abundance of both bacterial types continued to decline after the pandemic.

In contrast, *Legionella *infections significantly increased following the COVID-19 pandemic and continued to rise thereafter. Since the onset of COVID-19, reports have noted a decrease in the prevalence of *S. pneumoniae *[10], with mask-wearing suppressing droplet infections and reducing the overall number of infections.

Numerous studies have examined changes in bacterial identification due to the COVID-19 pandemic. Reviews indicate an overall increase in antimicrobial-resistant bacteria [11-13]. Reffat et al. reported a 24% increase in MRSA and a 14% decrease in 55 studies, while ESBL-producing *E. coli *showed no significant changes, based on data up to July 2023 [12]. Langford et al. reviewed 23 papers analyzing data up to 2022, with no changes in MRSA [14]. In Japan, a decline in MRSA has been observed [15]. The decline in *S. pneumoniae *infections is consistent with global trends [16]. Public health containment measures and awareness campaigns likely contributed to the reduction in pneumococcal infections, leading to a notable decrease in life-threatening invasive diseases worldwide [16]. A reduction in *P. aeruginosa *in outpatients has not yet been reported, although some studies have shown an increase in bloodstream infections due to *P. aeruginosa *in COVID-19 patients [17].

Reports on the increase of *Legionella* spp. during the pandemic remain inconclusive. However, water stagnation and poor building maintenance have been identified as factors increasing the risk of infection during COVID-19 lockdowns [18,19]. Additionally, studies have suggested an increased risk of *Legionella *infection during earthquakes [20,21].

Earthquake-related bacterial infections have been studied in earthquake-prone countries [5,22], but no studies have addressed changes in the number or frequency of bacterial infections post-earthquake. Our analysis showed that both antimicrobial-resistant and antimicrobial-susceptible bacteria continued to change in terms of incidence and frequency due to the COVID-19 pandemic. Notably, the significant reduction of MRSA and MRSE infections among outpatients persisted after the earthquake, while* Legionella *infections continued to increase in frequency. Earthquake-related damage to groundwater and hot springs may have facilitated the surface appearance of *Legionella*. Given the abundance of hot springs in Japan, the risk of *Legionella *infection during earthquakes must be considered. As hot springs and earthquakes are globally interrelated, this issue will likely continue to garner attention in future research.

The results of this survey differed from previous global data, possibly due to differences in national defense systems against the COVID-19 pandemic in Japan compared to other parts of the world [23]. In Japan, mask-wearing was rigorously maintained even after the pandemic subsided. Dabaja-Younis et al. in Israel suggested that enhanced infection protection measures and individual awareness during hospitalization for COVID-19 helped suppress drug-resistant bacterial infections [24]. At our hospital, infection prevention measures remain stringent, both individually and institutionally. These results could provide valuable insights for preventing bacterial infections in the future.

It is important to acknowledge several limitations of our study. First, as a retrospective observational analysis based on secondary data, we cannot draw causal conclusions from the trends observed. Second, since the data were collected from a single regional hospital, the generalizability of our findings to other populations or healthcare settings may be limited. To address these limitations, future research will involve multicenter data collection and more detailed patient-level analyses.

## Conclusions

The present study demonstrated that both the COVID-19 pandemic and the 2024 Noto Peninsula earthquake had a significant and lasting impact on bacterial infection dynamics within a hospital setting. The consistent decline in infections caused by MRSA, MRSE, *S. pneumoniae*, and *P. aeruginosa *suggests that sustained nonpharmaceutical interventions, especially mask-wearing and improved hygiene, can effectively reduce droplet- and contact-transmitted pathogens, even after the peak of a pandemic. On the other hand, the observed increase in *Legionella *infections highlights the vulnerability of environmental infection control following natural disasters. These findings underscore that while behavioral infection control measures can effectively suppress certain pathogens, disaster-induced infrastructural disruptions can facilitate the spread of others.

Together, these results emphasize the need for a dual infection control approach in Japan, where frequent seismic activity necessitates the concurrent implementation of both COVID-19-related infection prevention measures and countermeasures to address *Legionella *infections associated with earthquakes.
